# Development of mouse preimplantation embryos in space

**DOI:** 10.1093/nsr/nwaa062

**Published:** 2020-04-11

**Authors:** Xiaohua Lei, Yujing Cao, Baohua Ma, Yunfang Zhang, Lina Ning, Jingjing Qian, Liwen Zhang, Yongcun Qu, Tao Zhang, Dehong Li, Qi Chen, Junchao Shi, Xudong Zhang, Chiyuan Ma, Ying Zhang, Enkui Duan

**Affiliations:** State Key Laboratory of Stem Cell and Reproductive Biology, Institute of Zoology, Chinese Academy of Sciences, Beijing 100101, China; State Key Laboratory of Stem Cell and Reproductive Biology, Institute of Zoology, Chinese Academy of Sciences, Beijing 100101, China; College of Veterinary Medicine, Northwest A&F University/Key Laboratory of Animal Biotechnology, Ministry of Agriculture, Yangling 712100, China; State Key Laboratory of Stem Cell and Reproductive Biology, Institute of Zoology, Chinese Academy of Sciences, Beijing 100101, China; State Key Laboratory of Stem Cell and Reproductive Biology, Institute of Zoology, Chinese Academy of Sciences, Beijing 100101, China; State Key Laboratory of Stem Cell and Reproductive Biology, Institute of Zoology, Chinese Academy of Sciences, Beijing 100101, China; University of Chinese Academy of Sciences, Beijing 100049, China; State Key Laboratory of Stem Cell and Reproductive Biology, Institute of Zoology, Chinese Academy of Sciences, Beijing 100101, China; University of Chinese Academy of Sciences, Beijing 100049, China; State Key Laboratory of Stem Cell and Reproductive Biology, Institute of Zoology, Chinese Academy of Sciences, Beijing 100101, China; University of Chinese Academy of Sciences, Beijing 100049, China; Shanghai Institute of Technical Physics, Chinese Academy of Sciences, Shanghai 100049, China; Division of Ionizing Radiation, National Institute of Metrology, Beijing 100029, China; State Key Laboratory of Stem Cell and Reproductive Biology, Institute of Zoology, Chinese Academy of Sciences, Beijing 100101, China; State Key Laboratory of Stem Cell and Reproductive Biology, Institute of Zoology, Chinese Academy of Sciences, Beijing 100101, China; State Key Laboratory of Stem Cell and Reproductive Biology, Institute of Zoology, Chinese Academy of Sciences, Beijing 100101, China; State Key Laboratory of Stem Cell and Reproductive Biology, Institute of Zoology, Chinese Academy of Sciences, Beijing 100101, China; State Key Laboratory of Stem Cell and Reproductive Biology, Institute of Zoology, Chinese Academy of Sciences, Beijing 100101, China; State Key Laboratory of Stem Cell and Reproductive Biology, Institute of Zoology, Chinese Academy of Sciences, Beijing 100101, China

**Keywords:** space-flight, radiation, microgravity, preimplantation development, DNA damage, DNA methylation

## Abstract

The development of life beyond planet Earth is a long-standing quest of the human race, but whether normal mammalian embryonic development can occur in space is still unclear. Here, we show unequivocally that preimplantation mouse embryos can develop in space, but the rate of blastocyst formation and blastocyst quality are compromised. Additionally, the cells in the embryo contain severe DNA damage, while the genome of the blastocysts developed in space is globally hypomethylated with a unique set of differentially methylated regions. The developmental defects, DNA damage and epigenetic abnormalities can be largely mimicked by the treatment with ground-based low-dose radiation. However, the exposure to simulated microgravity alone does not cause major disruptions of embryonic development, indicating that radiation is the main cause for the developmental defects. This work advances the understanding of embryonic development in space and reveals long-term extreme low-dose radiation as a hazardous factor for mammalian reproduction.

## INTRODUCTION

The environment in space, unlike that on Earth, is characterized by microgravity and cosmic radiation, which have strong impacts on most biological systems [1,2]. Can humans or other animals reproduce in such an environment, and, specifically, how does the environment in space influence normal embryonic development? Over the past decades, numerous efforts have been made to study the impact on reproduction in space. Experiments with sea urchins, birds, fish and amphibians suggested that reproduction of non-mammalian species can still progress in the virtual absence of gravity [3–7]. Previous studies have attempted to examine the effects of space flight on mammalian mating and pregnancy by using unmanned biosatellites [8,9]. After being sent into orbit to allow mating, the mature male and female rats failed to produce offspring, although post-flight examinations showed that two out of five rats had signs of early pregnancy. Whether this is true for mammalian reproduction is still unclear due to the susceptibility of mammalian embryos to the cosmic environment during early development [10–12] and the technological limitations of cultivating a system that maintains the growth of embryos during spaceflight [13]. As a result, none of the attempts to investigate mammalian preimplantation embryonic development in space have been successful [14,15].

Technical difficulties and limited opportunities for space flight experiments in mammalian development encouraged some ground-based facilities for simulation of microgravity to be used to study the effect of microgravity on fertilization and preimplantation development in mammals. Ground-based experimental models that simulate weightlessness conditions revealed detrimental effects of microgravity on mouse preimplantation development, including delayed development and impaired blastocyst formation [16–18]. Nonetheless, these models cannot recapitulate the complex environment in space (including microgravity and cosmic radiation), and, as such, new technologies need to be developed to enable such research in space.

In the present study, we explored mouse preimplantation embryonic development during the spaceflight of China's SJ-10 recoverable satellite. We developed an automated mini incubator equipped with programmable controls for temperature, automatic micrography and fixation of samples for cultivating mouse embryos. We showed unequivocally that preimplantation mouse embryos can develop in space. The embryos developed through a series of cell divisions towards blastocoel morphology during spaceflight, but the rate of blastocyst formation and blastocyst quality were markedly reduced. Additionally, the cells in the embryo contain severe DNA damage, while the genome of the blastocysts developed in space is globally hypomethylated with a unique set of differentially methylated regions (DMRs). The developmental defects, DNA damage and epigenetic abnormalities can be largely mimicked by the treatment with ground-based low-dose radiation, indicating that cosmic radiation is the main cause for the developmental defects. This work advances the understanding of embryonic development in space and reveals long-term extreme low-dose radiation as a hazardous factor for mammalian reproduction.

## RESULTS

### Establishment of an automated culture system for mouse preimplantation development in space

**Figure 1.**
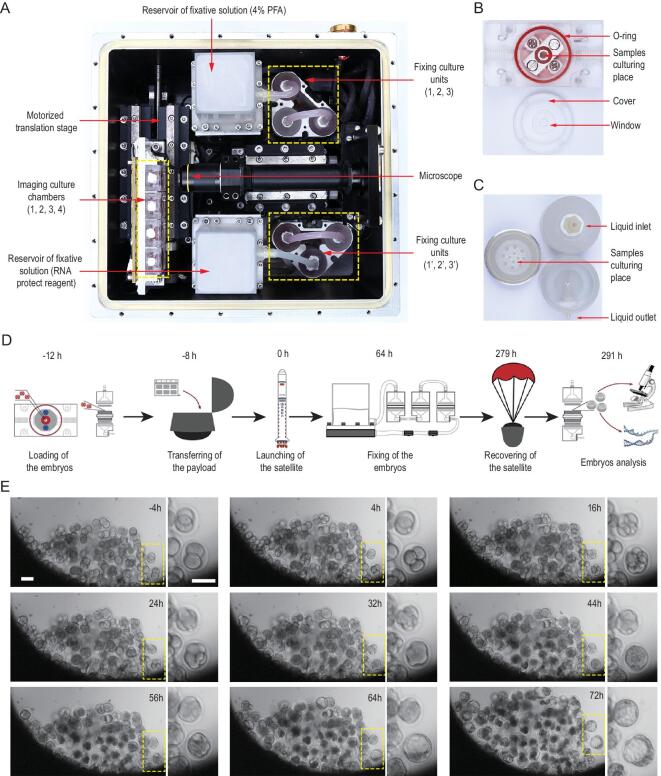
*In vitro* development of mouse pre-implantation embryos in space. (A) The embryonic culture incubator used in space experiments. The incubator consists of four imaging culture chambers (indicated by the yellow dotted line and the numbers 1, 2, 3 and 4), two groups of culture fixation units (yellow dotted lines), a microscope (red arrow), and two module reservoirs of fixative solutions (red arrow). This experimental apparatus provides temperature stability inside the incubator, the ability to replace culture medium and automatic image acquisition. (B) The imaging culture chamber was filled with gas-saturated medium, and it consisted of a sample culture cavity with a red O-ring and a cover with a window. (C) The fixation culture unit is a cylindrical perfusion chamber with a top part of the chamber, main body and bottom part of the chamber. (D) Timeline of the SJ-10 satellite space mission showing the time points for embryos loading, payload transferring, embryos fixation, sample recovery and arrival to the laboratory. (E) Representative time-lapse images of embryonic development in imaging culture chambers during spaceflight, with highlighted images showing key stages of pre-implantation development. Scale bars, 100 μm.

We developed an automated mini incubator to enable mouse early development in space that was equipped with programmable controls for temperature, automatic micrography and fixation of samples, for cultivating mouse embryos (Fig. 1A, and Fig. S1A and B). We first asked whether the growth of preimplantation mouse embryos in this mini incubator was feasible under sealed conditions (Fig. S2). To do so, we cultured frozen-thawed 2-cell embryos for 64 h under conventional culture (CC) in the microdroplet CO_2_ incubator or sealed culture (SC) in developed automated mini incubator conditions and compared the experimental outcomes. Under the CC conditions, most of those 2-cell embryos reached the morula/blastocyst stage. Likewise, the SC conditions allowed the 2-cell embryos to achieve a very similar developmental outcome (88.16% for CC vs 85.09% for SC) (Fig. S3A). Furthermore, 130 blastocysts from SC conditions and 90 blastocysts from CC conditions were transferred into pseudopregnant mice (9 to 12 embryos into one recipient female) to observe the potential to develop to full-term offspring (Fig. S3B). The recipients gave birth to 42 (SC) and 35 pups (CC), respectively. The rate of live offspring production from SC embryos (32.31%), albeit slightly lower than that from CC embryos (38.89%), was not significantly different (Fig. S3D). Additionally, the body weight (1.61 g vs. 1.58 g) and the sex of the pups (51% vs 47.6% female) were similar between SC and CC groups (Fig. S3E and F), while all of the pups were capable of growing to adulthood and delivering litters. Thus, the automated mini incubator allows the preimplantation mouse embryos to develop normally under the SC conditions and is therefore suitable for subsequent experimentation in spaceflight. Two automated mini incubators were developed, one for experiments in space and one on Earth with consistent composition.

The SJ-10 recoverable satellite was launched on 6 April 2016. It had an orbital attitude of ∼252 km, a microgravity level of 10^−4^−10^−6^g_0_ and an average dose of radiation of ∼0.15 mGy/day [19]. To assess mouse preimplantation embryonic development during spaceflight, 3400 morphologically normal mouse embryos at the 2-cell stage were placed into four imaging culture chambers (Fig. 1A and B) and six culture fixation units (Fig. 1A and C), respectively, 12 hours before launching of the satellite. The embryos in imaging culture chambers were monitored using microscopy to observe the morphology of live embryos every 4 hours, while the embryos in culture fixation units were either fixed with 4% paraformaldehyde (PFA) or treated with RNA protection reagents after 64-hour cultivation in orbit (Fig. 1D). Time-lapse imaging demonstrated that 2-cell embryos cultured in space underwent key stages of mammalian preimplantation development, including cleavage, compaction of blastomeres, cavitations and formation of expanded blastocysts (Fig. 1E and movie S1 to movie S4). However, the developmental defect of the embryo was significantly observed under the sealed culture conditions in space (SS) when compared with that under the SC conditions on Earth (movie S5 to movie S8).

### The rate of blastocyst formation and blastocyst quality are compromised in space

**Figure 2.**
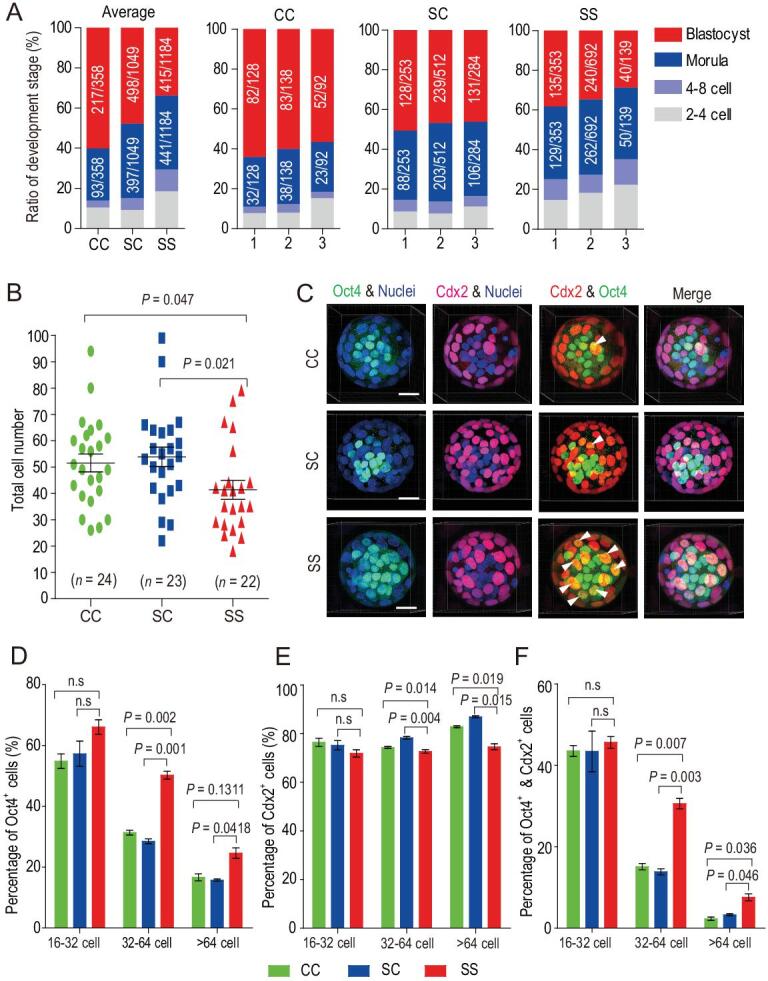
Embryonic development and blastocyst quality are compromised in space culture condition. (A) Analysis of embryonic development in space in fixation culture units after satellite return to Earth. Data represent means of all embryos acquired from three independent units of fixative (1: indicates unit 1; 2: indicates unit 2 and 3: indicates unit 3). CC: indicates conventional culture, SC: indicates sealed culture and SS indicates space sealed culture. (B) The total cell number in blastocysts under CC, SC and SS conditions calculated from 3D reconstruction analysis. Two-tailed Student's *t*-tests were used for statistical analysis. The SS group had significantly fewer cells than the CC (*P* = 0.047) and SC groups (*P* = 0.021). Each dot represents one embryo, and black bars indicate the mean cell number for each group. (C) Representative 3D images of Cdx2 and Oct4 immunofluorescence in blastocysts (>64-cell stage) developed under CC, SC and SS conditions. Oct4 stains the ICM (green), while Cdx2 stains the TE (red). Nuclei were stained with Hoechst33342 (blue). Arrowheads denote colocalization of Oct4 and Cdx2 (yellow). Scale bar, 20 μm. The percentage of Oct4-positive cells (D), Cdx2-positive cells (E) and double-positive cells (F) in CC, SC and SS embryos at the 16–32 cell, 32–64 cell and >64 cell blastocyst stages, respectively. The results are shown as the means ± SEM. *P* values are determined by Student's *t*-test (two-tailed); (SS vs. CC and SS vs.SC). n.s., not significant (*P* > 0.05).

A limitation of the time-lapse imaging analysis is the low number of observable embryos due to floatation, aggregation and a lack of accessibility. Thus, we further assessed the morphology and rate of development of the embryos in fixation culture units returned from space. Of the 1500 embryos loaded, 1184 were recovered and consisted of compacted morulas and expanded blastocysts (Fig. S4). Among them, the majority (856/1184, or 72.3%) developed into morula/blastocysts, but the ratio of the blastocyst-stage (34.3%) was significantly lower than that of the CC (60.24%, *P* = 0.0023) and the SC group (56.87%, *P* = 0.0024) (Fig. 2A), indicating impaired developmental ability. Thus, 2-cell mouse embryos can develop into blastocysts in space, but the ratio of embryos developed to the blastocyst stage is greatly compromised.

After assessing the rate of development, we examined the quality of the blastocysts developed in space and found that consistent with the reduced rate of embryonic development, the total number of cells in the blastocysts (41.5 cells) developed in space (i.e. the SS condition) decreased significantly when compared with that of the CC (51.6 cells) and the SC (53.9 cells) condition (Fig. 2B). We performed immunostaining of Cdx2 (a trophectodermal (TE) marker) [20,21] and Oct4 (an inner cell mass (ICM) marker) in blastocyst-stage embryos [22,23], allowing us to find an expression pattern in SS embryos that is highly distinguishable from that in the CC or SC embryos (Fig. 2C). During normal mouse embryonic development, Oct4 was widely expressed in both the inner and outer layers of cells in blastocysts with 16–32 cells, while Cdx2^+^ cells were mainly located in the outer layer (Fig. S5A). In the blastocysts with 32–64 cells, Oct4 expression was diminished in a subset of TE cells, while a few Oct4 and Cdx2 double-positive cells still existed (Fig. S5B). In blastocysts with >64 cells, Oct4 expression was restricted to cells in the ICM and was largely absent in Cdx2^+^ trophectoderm cells (Fig. S5C). Using the Imaris cell imaging software, we quantified the number of nuclei (blue), Oct4^+^ (green), Cdx2^+^ (red), and Oct4^+^ and Cdx2^+^ cells (yellow) in blastocysts developed under the CC, SC and SS conditions. Strikingly, there was a significant increase in the percentage of Oct4^+^ cells in SS embryos at both the 32–64 and >64 cell stages (Fig. 2D). In contrast, the percentage of Cdx2^+^ cells was reduced in SS embryos at these stages (Fig. 2E). In keeping with these findings, the percentage of Cdx2^+^/Oct4^+^ cells was significantly higher in the SS embryos than in the SC and CC embryos (Fig. 2F).

The increase in undifferentiated (i.e. Oct4^+^) cells and transitional Oct4^+^/Cdx2^+^ cells, and the concomitant decrease in lineage-specific Cdx2 cells suggest that cellular differentiation from the pluripotent state was compromised under the SS condition, consistent with earlier reports [17]. To further confirm this notion, we examined the epiblast (EPI) and primitive endoderm (PrE) cell fate specification in various types of embryos at the blastocyst stage by using the EPI-marker Nanog and the PrE-marker Gata6. In normal embryos under the CC and SC conditions, Nanog and Gata6 were co-expressed within the uncommitted ICM at the stage of <32 cells (Fig. S6A), but they displayed a ‘salt and pepper’ pattern at the stage of 32–64 cells (Fig. S6B). Notably, in the SS embryos, many Nanog-positive cells still expressed Gata6 in hatching blastocysts (Fig. S6C). Together, these results suggest that although the environment in space does not affect polarization and the establishment of the ICM and TE, growth and cellular differentiation are severely compromised in preimplantation mouse embryos.

### DNA damage and DNA methylome alterations in mouse embryos in space

**Figure 3.**
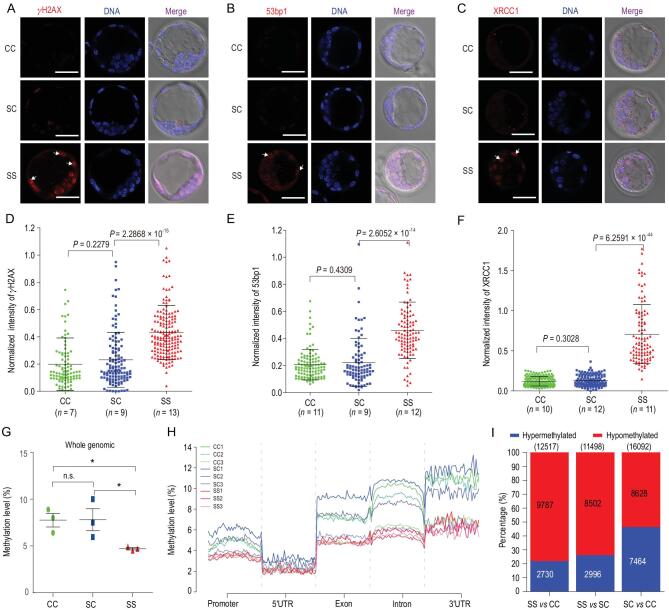
DNA damage and DNA methylome alterations in mouse embryos in space. (A–C) Representative images of CC, SC and SS blastocysts stained with γH2AX, 53bp1 and XRCC1 antibodies, respectively. Distinct staining patterns of cells were observed in blastocysts developed in space (white arrowheads). Scale bars, 50 μm. (D–F) Quantification of γH2AX (D), 53bp1 (E) and XRCC1 (F) immunofluorescence intensity normalized to DNA staining by Hoechst and analysed with ImageJ software. One imaging section was selected for each embryo for quantification, and the pooled data from embryos were plotted in the graphs. *n* = number of embryos. The results are shown as the means ± SEM. *P* values are from two-tailed unpaired Student's *t*-test. (G) The levels of genome-wide DNA methylation in CC, SC and SS blastocysts are shown. Three blastocysts were used for each group. The result are shown as the means ± SEM. Asterisks indicate the significance level of *P* < 0.05 (*t*-test). (H) Distribution of DNA methylation in the CpG context among various genomic element regions, including promoters, 5'UTR, exons, introns and 3'UTRs in CC, SC and SS blastocysts. The numbers denote the ID of specific blastocysts. (I) The number of differentially methylated regions (DMRs) in each pair-wise comparison of groups are shown. Blue and red bars indicate the proportion of hypermethylated and hypomethylated DMRs.

After revealing the developmental defects of SS embryos during development, we investigated the potential underlying mechanisms. Previous studies suggested that radiation in space can increase the frequency of DNA damage in *Drosophila Melanogaster* and human cells during spaceflight [24–26]. We therefore examined the formation of γH2AX and 53bp1 foci by using these two biomarkers for double-strand breaks (DSBs) of DNA repair [27,28]. In sharp contrast to the ground-cultured blastocysts, many blastomeres of the SS blastocysts were positive for γH2AX and 53bp1, showing stronger γH2AX and 53bp1 signals in the nuclei (Fig. 3A and B). In addition, experiments conducted using XRCC1, a maker for efficient repair of DNA single-strand breaks (SSBs) [29], showed that XRCC1 was localized in the nuclei of the SS blastocysts (Fig. 3C). Expectedly, quantification of fluorescence intensity confirmed a significant increase in γH2AX, 53bp1 and XRCC1 expression in SS blastocysts in comparison with the ground-cultured embryos (Figs 3D–F). These results indicate that the exposure of mammalian preimplantation embryos to the space environment causes severe DNA damage in the cells.

Epigenetic regulation such as DNA methylation plays a vital role in early mammalian embryonic development [30]. The developmental defects in SS embryos led us to ask whether they are linked to changes in DNA methylation during preimplantation development. We performed genome-wide DNA methylation profiling of individual blastocysts using bisulfite sequencing (BS-seq) [31]. Three expanded blastocysts from each group were collected and analysed (Fig. S7A). Methylome analysis showed that the percentage of global cytosine-guanine (CG) methylation in SS blastocysts (4.70%±0.2%) was significantly lower than that in CC (7.77%±1.3%) and SC (7.82%±2.0%) embryos (Fig. 3G and Fig. S7B and C), and the decrease was accompanied by a reduction in methylation density (Fig. S8). Additionally, we discovered a high extent of hypomethylation for DNA elements, including promoters, untranslated regions (UTRs), exons, introns and intergenic regions (Fig. 3H, and Fig. S9A–F) in the SS embryos compared with the CC and SC embryos. We further analysed DMRs, allowing us to identify 12 517 DMRs between the SS and CC conditions and 11 498 DMRs between the SS and SC conditions. There were fewer DMRs with high methylation between SS and CC (*n* = 2730) and between SS and SC (*n* = 2996) than between SC and CC (*n* = 7464), consistent with the low-level global methylation in SS blastocysts (Fig. 3I, and Fig. S7D). We then annotated overlapping DMRs with hypermethylation or hypomethylation (‘exclusive’ DMRs) for each group. Functional enrichment analyses indicated that the low-methylation exclusive DMRs in the SS blastocysts were related mainly to histone modifications, responses to radiation, regulation of chromosome organization, cytoskeleton organization and others (Fig. S7E and F), while high-methylation exclusive DMRs for CC blastocyst were associated with embryonic development, regulation of RNA metabolic processes and regulation of intracellular protein transport. These results suggest that in response to the environmental factors in space the genes responsible for histone modifications and radiation are hypomethylated, leading to their activation and subsequent repair of DNA damage.

### Effects of ground-based radiation on embryonic development

**Figure 4.**
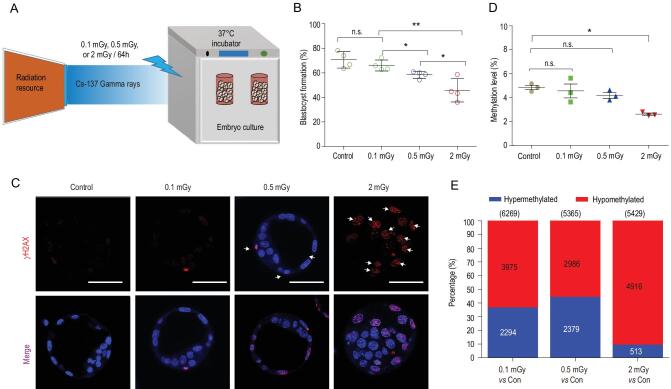
Effects of ground-based radiation on embryonic development. (A) To investigate the responses of mouse embryos to the accumulated low- doses of radiation, we irradiated 2-cell embryos with Cs-137 gamma at the doses of 0.1 mGy, 0.5 mGy and 2 mGy in ground-based experiments. (B) The percentage of 2-cell embryos that successfully developed to the blastocyst stage during *in vitro* cultivation with radiation exposure. Data are presented as the means ± SEM from four independent experiments. ^*^P < 0.05; ^**^P < 0.01; n.s., not significant (*P* > 0.05). (C) Representative fluorescence images of γH2AX in blastocysts that developed from 2-cell embryos with or without exposure to different doses of radiation for 64 h. Scale bars, 50 μm. (D) The level of genome-wide DNA methylation in blastocysts exposed to 0, 0.1, 0.5 and 2 mGy radiation. The results are shows as the means ± SEM. ^*^P < 0.05; n.s., not significant (*P* < 0.05) (*t*-test). (E) The number of differentially methylated regions (DMRs) in each pair-wise comparison of groups are shown. Blue and red bars indicate the proportion of hypermethylated and hypomethylated DMRs.

The mouse preimplantation embryos in space are exposed to two main environmental factors: radiation and microgravity. To ask what factors might have caused the developmental, genetic and epigenetic abnormalities in the embryos, we employed ground-based low-dose radiation to mimic the environment inside the SJ-10 satellite (cumulative dose of radiation: ∼0.15 mGy/day) (Fig. 4A). We found that most 2-cell embryos (66.01%) exposed to 0.1 mGy for 64 h can develop to the blastocyst stage. However, when the dose was increased to 0.5 mGy (which is equivalent to the dose in the satellite), only 58.44% of the embryos reached the blastocyst stage, and the percentage decreased to 45.69% when the dose was increased to 2 mGy (Fig. 4B and Fig. S10). Consistent with the developmental defects, we detected fluorescent foci positive for γH2AX in embryos exposed to 0.5 mGy or 2 mGy, with the foci more intense with 2 mGy exposure (Fig. 4C).

Next, we analysed genome-wide DNA methylation profiling in mouse embryos with radiation exposure (Fig. S11A and B), which, as expected, induces global changes in DNA methylation. There was a statistically significant decrease in genome-wide DNA methylation in embryos with 2 mGy exposure when compared with the control (Fig. 4D and Fig. S11C), but not with the 0.1 or 0.5 mGy exposure. Analysis of DNA-methylated genomic elements also revealed hypomethylation after exposure of embryos to 2 mGy radiation (Fig. S11D), including a reduction in the methylation of genomic elements (Fig. S12) and in the density of methylation distribution of CG (Fig. S13). We also examined DMRs between the control and the embryos with radiation, allowing us to identify 5429 DMRs between the control and 2 mGy group, of which 4916 (90.5%) were hypomethylated, and 5365 DMRs between control and the 0.5 mGy group, of which 2986 (55.7%) were hypomethylated (Fig. 4E, and Fig. S11E).

Additionally, genes with differential methylation were enriched in cellular processes such as responses to radiation, responses to stress and cytoskeletal organization, regulation of histone modifications, regulation of histone acetylation and regulation of protein ubiquitination (Fig. S11F and G). Disruption of these processes might cause developmental defects of embryos and reduced offspring birth.

To examine the long-term consequences of radiation, we determined whether the embryos with radiation exposure could develop to full-term mice. We transferred the irradiated and control blastocysts into pseudopregnant females at day three (Fig. S14A) and compared the birth rates. Although live offspring were obtained from embryos with radiation exposure (Fig. S14B), the overall rates of production from embryos with 0.5 mGy (21.07%) and 2 mGy exposure (7.45%) were strikingly lower than that from embryos without radiation exposure (32.61%) (Fig. S14B to D). Thus, mouse embryos exhibit hypersensitivity to extremely low doses of γ-radiation during preimplantation development *in vitro*.

### Effects of ground-based microgravity simulation on embryonic development

**Figure 5.**
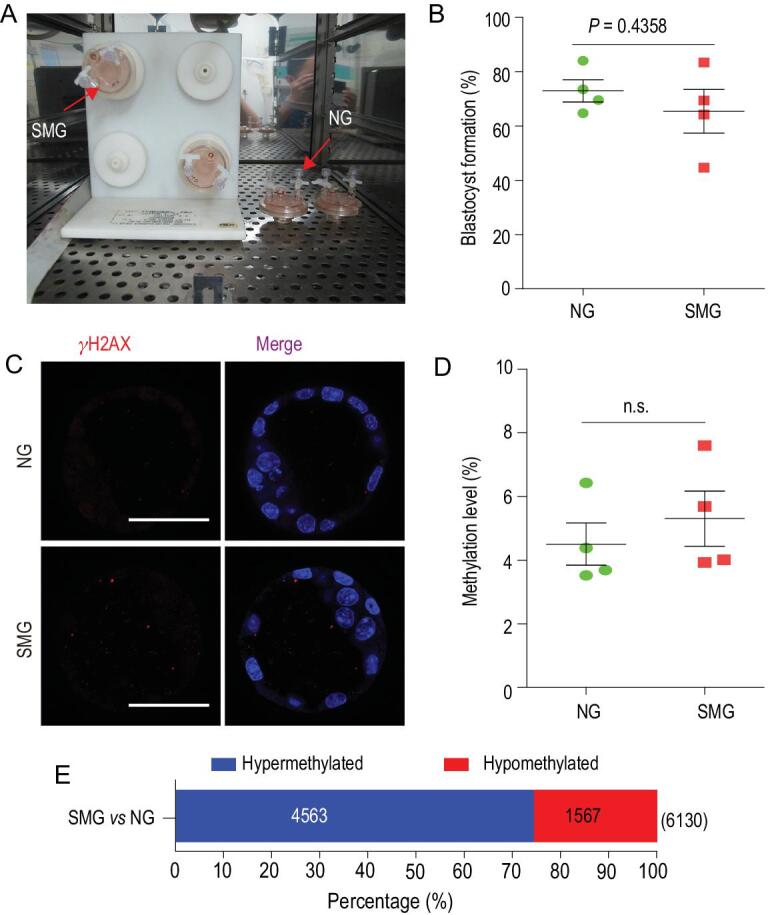
Effects of simulated microgravity on preimplantation embryonic development. (A) An image of the experimental apparatus, in which the embryos were inoculated into 10 mL of the culture vessel of RCCS for the simulated microgravity culture and static culture in CO_2_ incubators. (B) The rates of development of embryos to the blastocyst stages under normal gravity (NG) and simulated microgravity (SMG) conditions. Data are presented as the means ± SEM from four independent experiments (embryos analysed: *n* = 899 for NG; *n* = 886 for SMG). (C) Representative fluorescence images of γH2AX in blastocysts that were developed from 2-cell embryos exposed to NG and SMG conditions for 64 h. Scale bars, 50 μm. (D) The levels of genome-wide DNA methylation in NG and SMG blastocysts are shown. Data are presented as the means ± SEM from four blastocysts. (E) The number of differentially methylated regions (DMRs) between SMG and NG group are shown. Blue and red bars indicate the proportion of hypermethylated and hypomethylated DMRs. Throughout, a Student's *t*-test (two-tailed) was used for statistical analysis; n.s., not significant.

Having assessed the role of low-dose radiation in mouse embryonic development, we asked whether microgravity could also cause defects in preimplantation embryos. We cultured 2-cell mouse embryos in a rotary cell culture system (RCCS), which was developed by NASA for the purpose of simulated microgravity (SMG), and subsequently examined the effects (Fig. 5A). We found that most 2-cell embryos (65.4%) exposed to SMG for 64 h could develop to the blastocyst stage, without statistically significant differences (65.4% vs 72.9%) when compared with normal gravity (NG) (Fig. 5B and Fig. S15). Furthermore, we failed to observe any differences in DNA damage between control blastocysts and those developed with SMG (Fig. 5C). We also investigated genome-wide DNA methylation profiling in mouse blastocysts developed with SMG exposure (Fig. S16A). The heatmap of the percentage of DNA methylation at differentially methylated CpG sites for each blastocyst showed a similar methylation pattern (Fig. S16B). No statistically significant difference was found between blastocysts with SMG and with NG in the genome-wide DNA methylation level (Fig. 5D and Fig. S16C), including the distribution of DNA methylation in the CpG context among various DNA elements (Fig. S16D and Fig. S17) and in the density of methylation distribution of CG in chromosomes (Fig. S18). Intriguingly, 6130 DMRs were identified between the SMG and the NG group and were composed of 4563 DMRs with hypermethylation and 1567 DMRs with hypomethylation (Fig. 5E). Thus, a lower proportion of hypomethylated DMRs was discovered.

## DISCUSSION

In this study, we have developed an automated mini incubator equipped with programmable controls for temperature, automatic micrography and fixation of samples for cultivating mouse embryos. This device enables us to examine the development of 2-cell embryos during spaceflight in real time. Our results show unequivocally that preimplantation mouse embryos can develop in space, although the rate of blastocyst formation and blastocyst quality are compromised. At the mechanistic level, the cells in the embryos contain severe DNA damage. Additionally, the genome of the blastocysts developed in space is globally hypomethylated with a unique set of DMRs that are associated with embryonic development, regulation of RNA metabolic processes and regulation of intracellular protein transport. The developmental, genetic and epigenetic abnormalities can be largely mimicked by the use of ground-based low-dose radiation. However, we fail to observe significant differences in embryonic development, DNA damage or global hypomethylation under ground-based simulated microgravity conditions when compared with normal gravity conditions. These results demonstrate that radiation is the main factor contributing to the embryonic developmental defects, which provides a novel insight into the reproductive hazards of space radiation or long-term exposure to extreme low-dose radiation.

Previously, pregnant female rats were sent to space to explore whether weightlessness affects embryonic development and the parturition of mammals. Although the exposure to low Earth orbit during mid to late gestation does not cause observable detriments in fetal development, the rats during early gestation fail to produce offspring [9,32]. Thus, the exposure of early-stage embryos to the space environment is likely the cause of the loss of pregnancy [12]. Indeed, several studies have been performed to assess the *in vitro* development of mammalian preimplantation embryos in space, but no successful embryonic development has been observed. It is thus unclear to date that the failures of early embryonic development of mammals are attributed to technological shortcomings or space environment factors including radiation and microgravity [33].

Radiation has been shown to induce epigenetic alterations in DNA methylation modifications and global hypomethylated DNA [34]. We also found that the exposure of blastocysts developed in a space environment display marked DNA hypomethylation and contain DMRs exclusively related to histone modifications [35], regulation of histone H4 acetylation [36], regulation of chromosome organization, and response to radiation [37]. These results are consistent with those from a previous SJ-10 space study demonstrating low methylation levels in the genome of Arabidopsis seedlings under the cosmic environment [38]. In our ground-based radiation experiments, we found that whole-genome hypomethylation occurs with 2 mGy exposure, but not with 0.1 or 0.5 mGy exposure, suggesting dose-dependent epigenetic changes during preimplantation in response to low-dose ionizing radiation.

Previous studies reported negative effects of microgravity simulation with a 3D clinostat on mammalian preimplantation development, including delayed development and impaired blastocyst formation [16–18]. In the current study, we used a rotating wall vessel (RWV) to establish ground-based SMG and did not find statistically significant differences in blastocyst formation between control embryos and those cultured with SMG. In addition, we did not observe significant differences in DNA damage or DNA hypomethylation at the genome level. The discrepancies might also be attributed to the difference in artificial microgravity in ground-based studies, embryo culture methods and the developmental stage of embryos exposed to SMG between these studies.

Although microgravity alone does not affect embryonic development or DNA methylation, low-dose radiation is unlikely to be the only factor in space that causes developmental defects for the following reasons. First, the rate of blastocyst formation in the space environment is significantly lower than that in embryos with 0.5 mGy exposure (34.3% vs 58.4%), while the DNA damage in the SS embryos is greater than in the embryos exposed to 0.5 mGy (Fig. 3A and Fig. 4C). Additionally, although the DNA damage with 2 mGy exposure is similar to that of space embryos, the rate of blastocyst formation is higher than that for the SS embryos (45.69% vs 34.3%). The inability of radiation alone to fully mimic the effects of the space environment on mouse embryonic development raises the possibility of synergism between microgravity and radiation in the induction of impaired cellular growth and DNA damage [39,40].

In conclusion, we show, for the first time, that preimplantation mouse embryos can develop into blastocysts during spaceflight and that the environment in space impairs embryonic development and the quality of the blastocysts, causing DNA damage and epigenetic abnormalities. Our findings also suggest that cosmic radiation is the main factor for the developmental defects in the embryos developed in space. Additionally, we revealed, for the first time, the detrimental effects of extremely low-dose γ radiation on mammalian early embryonic development. As such, this environmental factor should be considered with a keen interest in future clinical settings.

## METHODS

Methods were described in detail in the Supplementary Materials.
